# Supplementary material for: Evaluation of Antifungal Selective Toxicity Using Candida glabrata ERG25 and Human SC4MOL Knock-In Strains
Source: J Fungi (Basel). 2023 Oct 20;9(10):1035. doi: 10.3390/jof9101035 (PMC10607794; doi:10.3390/jof9101035)
Supplement: Supplementary file 1 [file jof-09-01035-s001.zip › jof-2664561-supplementary.pdf]

Supplementary data

```
MSMO1   ATGGCAACAAATGAAAGTGTCAGCATCTTTAGTTCAGCATCCTTGGCTGTGGAATATGTA 60
hERG25  ATGGCCACCAATGAATCTGTATCCATCTTTAGCTCAGCATCATTAGCAGTTGAATATGTT 60
        ***** ** ******  ***   ********* ******** ** ** ** ********

MSMO1   GATTCACTTTTACCTGAGAATCCTCTGCAAGAACCATTTAAAAATGCTTGGAACTATATG 120
hERG25  GATAGTCTACTGCCTGAAAATCCACTGCAAGAACCCTTCAAGAATGCTTGGAATTACATG 120
        ***   **  * ***** ***** *********** ** ** *********** ** ***

MSMO1   TTGAATAATTATACAAAGTTCCAGATTGCAACATGGGGATCCCTTATAGTTCATGAAGCC 180
hERG25  TTGAACAATTACACGAAATTTCAGATAGCTACTTGGGGTTCTCTAATAGTTCACGAAGCG 180
        ***** ***** ** ** ** ***** ** ** ***** ** ** ******** *****

MSMO1   CTTTATTTCTTATTCTGTTTACCTGGATTTTTATTTCAATTTATACCTTATATGAAAAAA 240
hERG25  TTATATTTCTTGTTTTGTTTGCCAGGGTTCTTATTCCAATTCATCCCCTATATGAAGAAA 240
         * ******** ** ***** ** ** ** ***** ***** ** ** ******** ***

MSMO1   TACAAAATTCAAAAGGATAAGCCAGAGACATGGGAAAACCAATGGAAGTGTTTCAAAGTT 300
hERG25  TACAAGATTCAGAAGGATAAACCAGAAACTTGGGAAAATCAATGGAAATGCTTTAAAGTG 300
        ***** ***** ******** ***** ** ******** ******** ** ** *****

MSMO1   CTTCTCTTTAATCACTTCTGTATCCAGCTGCCTTTGATTTGTGGAACCTATTATTTTACA 360
hERG25  TTGCTTTTCAACCACTTTTGCATTCAATTGCCACTTATCTGTGGTACATACTACTTTACC 360
         * ** ** ** ***** ** ** **  ****  * ** ***** ** ** ** *****

MSMO1   GAGTATTTCAATATTCCTTATGATTGGGAAAGAATGCCAAGATGGTATTTTCTTTTGGCA 420
hERG25  GAATACTTTAACATTCCGTATGATTGGGAAAGAATGCCAAGATGGTATTTCCTGTTAGCA 420
        ** ** ** ** ***** ******************************** ** ** ***

MSMO1   AGATGCTTTGGTTGTGCAGTCATTGAAGATACTTGGCACTATTTTCTGCATAGACTCTTA 480
hERG25  AGATGCTTTGGATGTGCTGTTATCGAAGATACGTGGCATTACTTTCTACACAGATTATTA 480
        *********** ***** ** ** ******** ***** ** ***** ** *** * ***

MSMO1   CACCACAAAAGAATATACAAGTATATTCATAAAGTTCATCATGAGTTTCAGGCTCCATTT 540
hERG25  CATCATAAAAGGATCTACAAGTATATTCACAAAGTCCATCATGAGTTTCAAGCTCCATTT 540
        ** ** ***** ** ************** ***** ************** *********

MSMO1   GGAATGGAAGCTGAATATGCACATCCTTTGGAGACTCTAATTCTTGGAACTGGATTTTTC 600
hERG25  GGAATGGAGGCTGAGTATGCCCATCCTTTGGAAACATTGATACTTGGTACAGGCTTCTTC 600
        ******** ***** ***** *********** **  * ** ***** ** ** ** ***

MSMO1   ATTGGAATCGTGCTTTTGTGTGATCATGTAATTCTTCTTTGGGCATGGGTGACCATTCGT 660
hERG25  ATTGGCATTGTGTTGTTATGTGACCATGTCATATTGTTGTGGGCATGGGTTACTATAAGG 660
        ***** ** *** * ** ***** ***** **  *  * *********** ** **  *

MSMO1   TTATTAGAAACTATTGATGTCCATAGTGGTTATGATATTCCTCTCAACCCTTTAAATCTG 720
hERG25  TTACTTGAAACAATTGACGTACATAGTGGTTATGACATTCCTCTGAATCCTTTGAATCTA 720
        *** * ***** ***** ** ************** ******** ** ***** *****

MSMO1   ATCCCTTTCTATGCTGGTTCTCGGCATCATGATTTCCACCACATGAACTTCATTGGAAAC 780
hERG25  ATTCCGTTTTATGCGGGATCTAGACACCATGATTTCCACCATATGAACTTTATAGGTAAC 780
        ** ** ** ***** ** *** * ** ************** ******** ** ** ***

MSMO1   TATGCTTCAACATTTACATGGTGGGATCGAATTTTTGGAACAGACTCTCAGTATAATGCC 840
hERG25  TACGCTTCCACATTTACTTGGTGGGATCGTATTTTCGGTACTGATTCGCAATATAATGCC 840
        ** ***** ******** *********** ***** ** ** ** ** ** *********

MSMO1   TATAATGAAAAGAGGAAGAAGTTTGAGAAAAAGACTGAATAA                   882
hERG25  TATAACGAGAAAAGAAAGAAATTTGAGAAGAAAACCGAATAA                   882
        ***** ** ** ** ***** ******** ** ** ******
```

Figure S1. DNA alignment (Codon Conversion). The base sequences of human *ERG25* (*SC4MOL*) and human *ERG25* were converted to *the S. cerevisiae* codon (*hERG25*). Homologous sequences are denoted by asterisks (*).

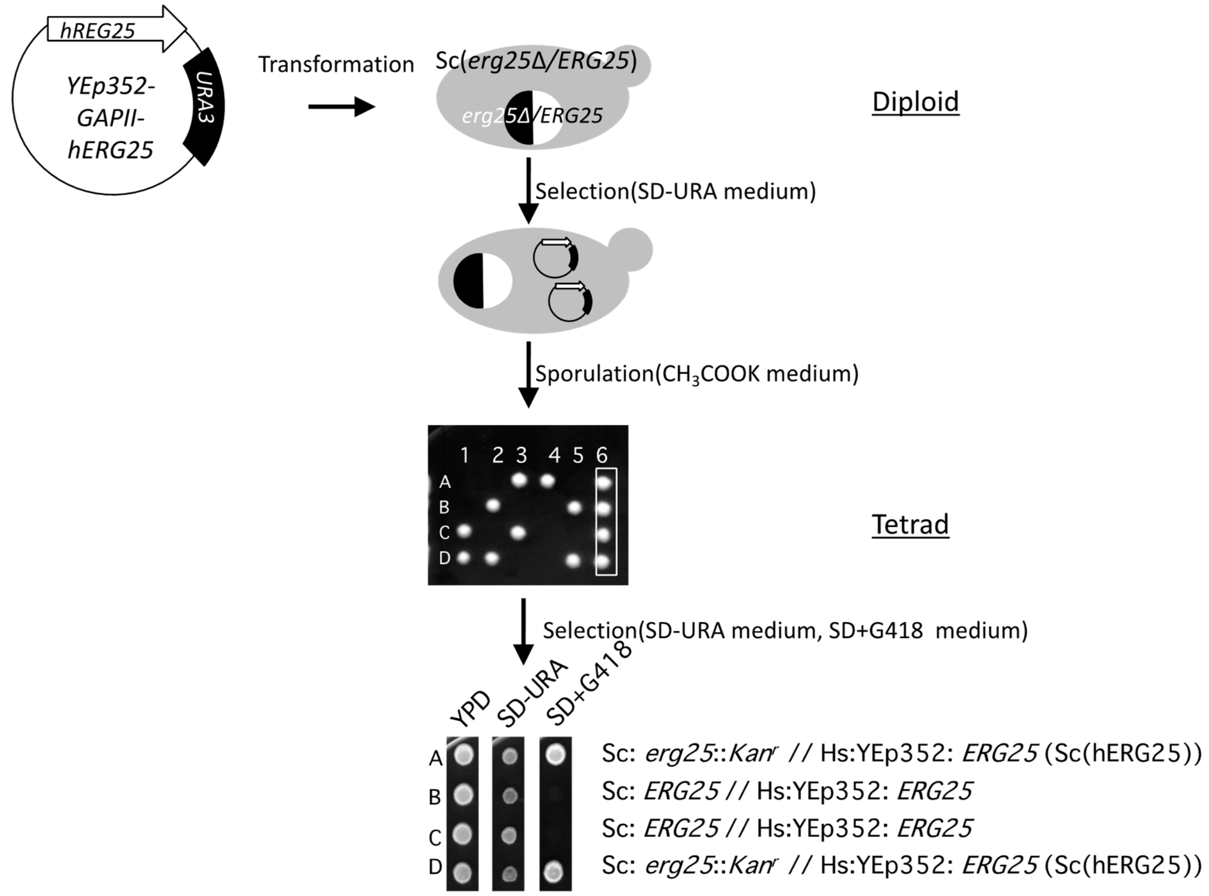

Figure S2. Summary of the mutational screen for *hERG25* variants complementing the loss of the yeast ortholog *ERG25*. Tetrad assay. All four strains derived from strain #6 possessed the hERG25p expression plasmid. The medium was selected. The four strains obtained from #6 were confirmed to grow on a selective medium, and A and D were found to be the strains that we needed.

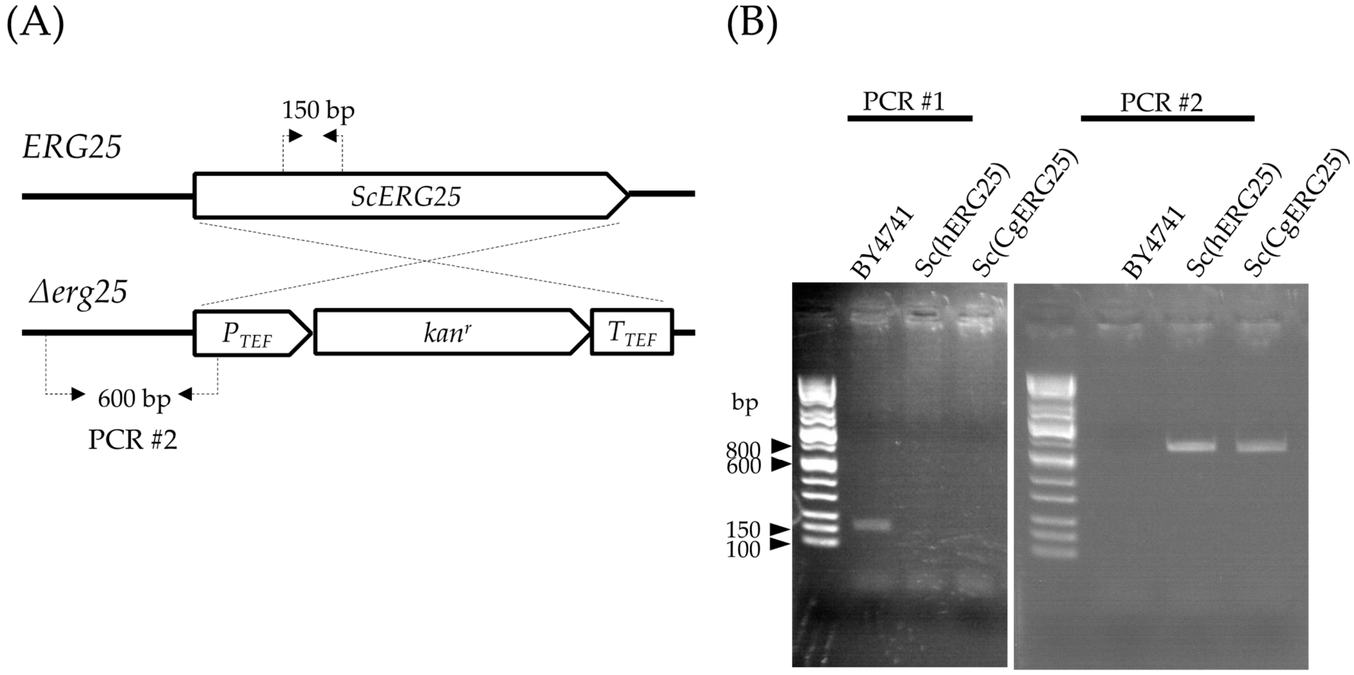

(C)

Primer name	Primer sequence	PCR
Sc_ScERG25rtF	TTTCTTGGTCGAGGCCATC	#1
Sc_ScERG25rtR	GGTGAGCCCAGTAATGCCA	#1
ScERG25-F1	ACTACCACTGCCTCCCTTCG	#2
TEF-R2	AGGGTATTCTGGGCCTCCATGT	#2

Figure S3. PCR for confirmation of *S. cerevisiae ERG25* deletion. (A) In PCR #1, the primer pair is situated within the ORF of *S. cerevisiae ERG25*, resulting in the amplification of a 150 bp PCR product. In the *ERG25*-deletion strain, the P_{TEF}-Kanr-T_{TEF} DNA cassette is replaced with *ScERG25* through homologous recombination. The PCR #2 primer pair is positioned upstream of the *ScERG25* on chromosome and at the P_{TEF} site of the cassette, and it amplifies a 600 bp PCR product. (B) The 150-bp band from PCR #1 was found in BY4741 but not in Sc(hERG25) and Cg(CgERG25). In PCR #2, a 600-bp band was found for Sc(hERG25) and Cg(CgERG25), while it was not in BY4741. (C) The base sequences of the primers used in PCR #1 and PCR #2 are shown.

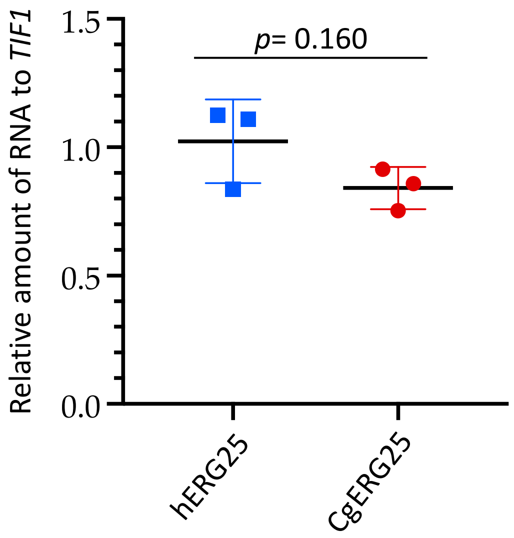

List of primers used in this experiment.

Primer name	Primer sequence
hERG25r-F1	GCAAGATGCTTTGGATGTGCT
hERG25-R1	CATACTCAGCCTCCATTCCA
CgERG25-F1	CAAAGAATTCATGTCTGCCGT
CgERG25-R1	TTACCGATGTTGGCCAAGGTC
ScTEF1rtF	ATTGGTACTGTGCCAGTCGG
ScTEF1rtR	CGTTGTCACCTGGAACACCT

Figure S4. Relative amount of RNA. Sc(hERG25) and Sc(CgERG25) strains were grown in minimal medium at 28 ℃ overnight. Cells were collected by centrifugation at 4 ℃. Total RNA was extracted using the ISOGEN (nippongene, Japan). ReverTra Ace® qPCR RT Master Mix (Toyobo, Japan) used to synthesize cDNAs.The amount of mRNA for each gene was determined by quantitative real-time PCR (qRT-PCR) on a LightCycler® 96 System (Roche Diagnosis, Germany) with SYBR Green detection using the Thunderbird SYBR qPCR mix (Toyobo). PCR conditions were as follows: pre-denaturation at 95 ℃ for 30 s, followed by 40 cycles of denaturation at 95 ℃ for 5s and annealing /extension at 60 ℃ for 1 min. mRNA levels were normalized to that of *TEF1*, a housekeeping gene that encodes elongation factor 1. The bars in the graph represent the average and standard deviation.

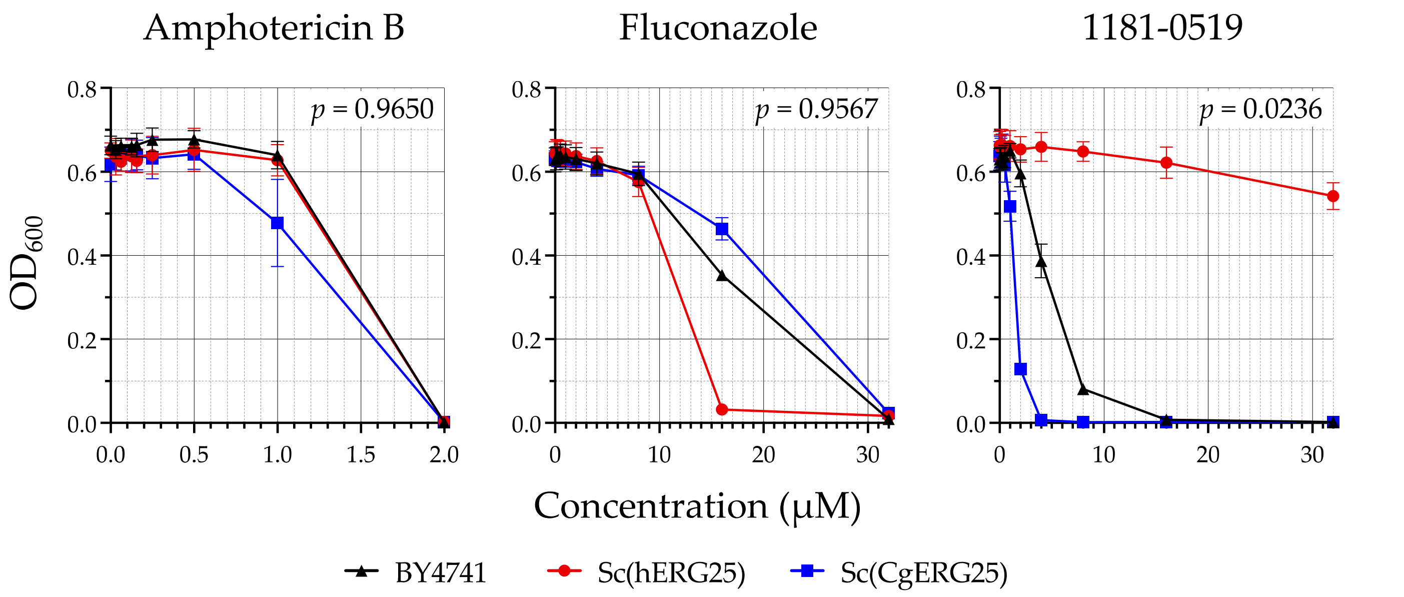

Figre S5. Antifungal susceptibility of knock-in strains. 100 μL of SD medium including uracil and leucine was added to each well of a 96-well microplate and incubated at 28 °C for 48 hours without shaking. The Y-axis and X-axis represent the OD_{600} and the concentration of each drug, respectively. Data are presented as the average of three replicates, with error bars indicating the avreage and the standard deviation. p-values represent the result of a one-way ANOVA.

(A)

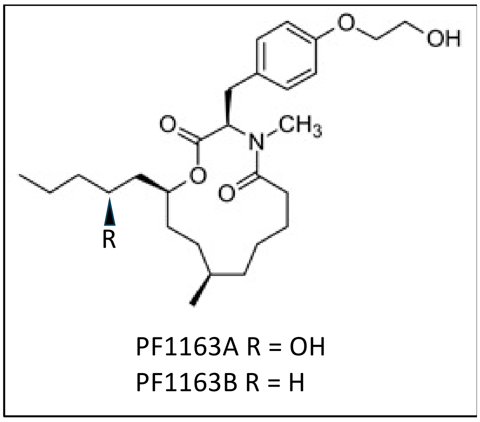

(B)

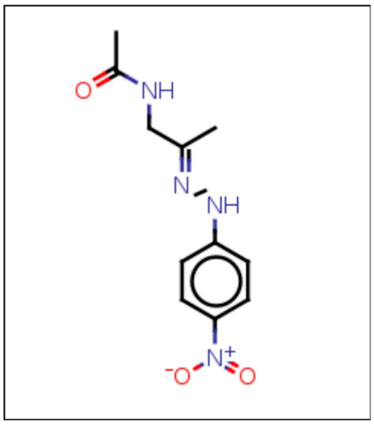

Figure S6. Molecular structure of two different types of Erg25p inhibitor. (A) Nose, H. *et al.* PF1163A and B, new antifungal antibiotics produced by *Penicillium* sp. I. Taxonomy of producing strain, fermentation, isolation and biological activities. *J. Antibiot. (Tokyo)* 53, 33–37(2000). (B) 1181-0519: N-[(2E)-2-[(4-nitrophenyl) hydrazinylidene]propyl]acetamide.

Lee, A. Y. *et al.* Mapping the Cellular Response to Small Molecules Using Chemogenomic Fitness Signatures. *Science* **344**, 208–211 (2014).

```
Homo_sapiens ------MATNESVSIFSSA---SLAVEYVDSLLPENPLQEPFKNAWNYMLNNYTKFQIAT 51
C.glabrata   ---MSAVFNNATLTDLVREDTYFKTLANIGKFQPQLNFMEQYWAAWYTYMNNDV--LATG 55
S.cerevisiae ---MSAVFNNATLSGLVQASTYSQTLQNVAHYQPQLNFMEKYWAAWYSYMNNDV--LATG 55
C.albicans   MSSISNVYHD--YSSFSNATTFSQVYQNFNQ-LDNLNVFEKLWGSYYYYMANDL--FATG 55
                   :  :   : :        .   .     :  . *    ::   : *      :

Homo_sapiens WGSLIVHEALYFLFCLPGFLFQFIPYMKKYKIQKDKPETWENQWKCFKVLLFNHFCIQLP 111
C.glabrata   LMFFLLHEFMYFFRCLPWFIIDQIPYFRKWKLQPTKIPSTKEQLYCLKAVLLSHFLVEAI 115
S.cerevisiae LMFFLLHEFMYFFRCLPWFIIDQIPYFRRWKLQPTKIPSAKEQLYCLKSVLLSHFLVEAI 115
C.albicans   LLFFLTHEIFYFGRCLPWAIIDRIPYFRKWKIQDEKIPSDKEQWECLKSVLTSHFLVEAF 115
                :: ** :**  ***  ::: ***::::*:*  *  : ::*  *:* :* .** ::

Homo_sapiens LICGTYYFTEYFNIPYDWERMPRWYFLLARCFGCAVIEDTWHYFLHRLLHHKRIYKYIHK 171
C.glabrata   PIWTFHPMCEKLGITVEVP-FPSIKKMSLEIALFFVLEDMWHYWAHRLFHYGVFYKYIHK 174
S.cerevisiae PIWTFHPMCEKLGITVEVP-FPSLKTMALEIGLFFVLEDTWHYWAHRLFHYGVFYKYIHK 174
C.albicans   PIWFFHPLCQKIGISYQVP-FPKITDMLIQWAVFFVLEDTWHYWFHRGLHYGVFYKYIHK 174
              *   : : : :.*  :   :*    :  .     *:** ***: ** :*:  :******

Homo_sapiens VHHEFQAPFGMEAEYAHPLETLILGTGFFIGIVLLC-----DHVILLWAWVTIRLLETID 226
C.glabrata   QHHRYAAPFGLSAEYAHPLETMSLGFGTVGMPILYVMYTGNLHLFTLCVWITLRLFQAVD 234
S.cerevisiae QHHRYAAPFGLSAEYAHPAETLSLGFGTVGMPILYVMYTGKLHLFTLCVWITLRLFQAVD 234
C.albicans   QHHRYAAPFGLAAEYAHPVEVALLGLGTVGIPIVWCLITGNLHLFTVSIWIILRLFQAVD 234
              **.: ****: ****** *.  ** * .   ::        *:: :  *: :**::::*

Homo_sapiens VHSGYDIPLNPLNLIPFYAGSRHHDFHHMNFIGNYASTFTWWDRIFGTDSQYNAYNEKRK 286
C.glabrata   SHSGYDFPWSLNKFLPFWAGAEHHDLHHHYFIGNYASSFRWWDYCLDTESGPEAKVAREE 294
S.cerevisiae SHSGYDFPWSLNKIMPFWAGAEHHDLHHHYFIGNYASSFRWWDYCLDTESGPEAKASREE 294
C.albicans   AHSGYEFPWSLHNFLPFWAGADHHDEHHHYFIGGYSSSFRWWDFILDTEAGPKAKKGRED 294
              ****::*  . :::**:**: *** **  ***.*:*:* ***  :.*::  :*   :..

Homo_sapiens KFEKKTE--------                                              293
C.glabrata   RMKSKAEQKAKKTN-                                              308
S.cerevisiae RMKKRAENNAQKKTN                                              309
C.albicans   KVKQNVEKLQKKNL-                                              308
             :.:...*
```

Figure S7: Erg25p Alignment. The primary structures of Homo sapiens (uniport: Q15800), *C. glabrata* (uniport: Q6FMX4), *S. cerevisiae* (uniport: P53045), and *C. albicans* (uniport: O59933) were aligned using Clustal Omega (https://www.ebi.ac.uk/Tools/msa/clustalo/). Alphabetic characters represent single letters of amino acid residues. The numbers on the right-hand side indicate the serial amino acid number, and the following symbols indicate amino acid residues that are completely conserved in the sequence '*', very well-conserved amino acid residues ':', conserved amino acid residues '.' are marked with signs. The box marks the region corresponding to the three histidine motifs conserved throughout eukaryotes in ERG25p. These motifs are iron-binding sites that are hypothesized to be important for enzyme function.

Table S1. List of primers used in this study.

Primer name	Primer sequence
hERG25-F1	GAATTCATGGCCACCAATGAATCTG
hERG25-R1	GTCGACTTATTCGGTTTTCTTCTCA
YEp352-GAPII-F	TGAGAAGAAAACCGAATAAGTCGACGTGAATTTACTTTAAATCTT[illegible]
YEp352-GAPII-R	CAGATTCATTGGTGGCCATGAATTCTTTGTTTGTTTATGTGTGTTTA
CgERG25-F1	ACACACATAAACAAACAAAGAATTCATGTCTGCCGTTTTCAACAA
CgERG25-R1	AGATTTAAAGTAAATTCACGTCGACTCAATTGGTCTTCTTAGCCT
YEp352-GAPII-F3	GTGAATTTACTTTAAATCTTGCATTT
YEp352-GAPII-R3	TTTGTTTGTTTATGTGTGTTTATTCG
ScTDH3-F1	CCAACCATCAGTTCATAGGT
YEp351(352)-R	CAACTGTTGGGAAGGGC
Sc_ScERG25rtF	TTTCTTGGTCGAGGCCATC
Sc_ScERG25rtR	GGTGAGCCCAGTAATGCCA
ScERG25-F1	ACTACCACTGCCTCCCTTCG
TEF-R2	AGGGTATTCTGGGCCTCCATGT
h_ScERG25rtF	GCAAGATGCTTTGGATGTGCT
h_ScERG25rtR	GCATACTCAGCCTCCATTCCA
pq13537(ERG25)R82	CTTACCGATGTTGGCCAAGGTC
